# Anarchism in Physic

**Published:** 1892-05-07

**Authors:** 


### The Doctor's Armchair.

### ANARCHISM IN PHYSIC.

It will, perhaps, startle a good many readers, even among doctors, to be seriously told that of all revolutionaries no class of men have shown themselves so anarchical as the members of the medical profession. The history of the *Ars Medicinæ* is one long record of jumping to opposite extremes. In pathology and in treatment the rule has always been the same. One generation has been made up of humoralists, the next of solidists; one age has lived and died in the faith of phlebotomy, the next has banned phlebotomy with that most tremendous of all anathemas—the anathema of absolute indifference and contempt.

A too great readiness to change one's mind is not a mark of intellectual strength. That particular weakness cannot be legitimately charged against medical men. But it is equally an evidence of defective mental capacity to change one's mind with great energy and completeness on very inadequate grounds; and it cannot be denied that at least former generations of medical men have turned pathological and therapeutic somersaults for no other reason than popular clamour. Even now there are many who firmly believe that bloodletting is one of the most effective of all the therapeutic resources we have, in certain well-understood cases. But who would dare to propose to let blood at the bedside of any except a poor patient or an inmate of a hospital? In the case of phlebotomy popular feeling is so much stronger than the courage of medical science that an admirable method of treatment for a certain class of cases has gone entirely out of use.

At the present time the medical mind is in a state of almost complete chaos, the result of methods and pronouncements which, if they had been initiated in politics, would have been considered nothing less than revolutionary and anarchical. As examples of what is meant, it is sufficient to name Koch's treatment of tuberculosis, and modern bacillary pathology. If Koch had been but a little more successful than he was, or if accident had favoured him as much as it has favoured certain well-known "quacks," there is no doubt that ninety-nine medical men in every hundred would have incontinently deserted all their old methods of dealing with phthisis, and gone over *per saltum* to the irrational heresy of Berlin. And why not? will ask a chorus of medical and popular voices? Why not? Is it not evident to every rational man that such an abrupt and unreasoning transference of practical faith from old methods to new is a tacit confession that in the estimation of their practitioners the old methods are little better than worthless? That would be a terrible confession to make. It is impossible for us to imagine English lawyers abandoning their jurisprudence for newly-discovered methods in a body, or still less the Christian clergy going over *en masse* to Buddhism or Mohammedanism.

Whatever the "young lions" of the physiological laboratories may think to the contrary, there is such a thing as a rational conservatism in medicine, a conservatism which contains every element of solid mental and clinical worth. A clergyman, who is also a scientific medical man, said to the writer the other day, "There is no living physician in this or any other country who can be compared with Hippocrates for all-round intellectual capacity." A statement like that, if made in an assemblage of medical students, would be greeted with roars of stupid laughter; but if it were made at a gathering of philosophic, cultured, and experienced physicians, it would be considered with serious and self-questioning gravity. It will not be denied that there have been giants in past times in other walks of life than medicine. Is it such a foolish thing to suppose there may have been giants in medicine too? Is not, indeed, the foolishness all the other way? What modern poet would not be content if the work and fame of Homer were his own? What nineteenth century moralist and philosopher would not be proud of the reputation of Socrates, even if it led him to the same want of appreciation and the same end? But Homer was not the only great poet, by many, who lived before the dawn of our era; nor was Socrates the only original thinker and reasonable philosopher. Hippocrates was a great physician; and he was only one of many. Compared with him the majority of modern practitioners, even of consideration and name, are but children in judgment and intellectual capacity.

There are two things which a physician of mature age must value and stand by if he be a man of any real intellect; and the two are the reasoned conclusions of his scientific studies, and the accumulated results of his own experience. These represent his gathered wealth, his safe investments. He lives by them at the bedside from day to day. The new things he reads of are at first, and ought to be to him, like the more or less promising prospectuses of new commercial companies. As a scientific man he has scientific resources to invest. He will therefore study the new things, the new pathologies and methods of bedside work; he will also experiment tentatively with them on a small scale and when safe opportunities offer; safe, that is, for his patients as well as for himself. But will he throw away in a moment all the reasoned conclusions of his scientific studies and all the accumulated results of his experience, and incontinently hand over all his patients to the tender mercies of methods that are entirely new and that are vouched for by only one man, or even by half-a-dozen men? He may, if he be an inconsequent fledgling. But if he be a man of any real sense and capacity he will stand by his proved methods in his regular work, and not trust the new at all until he has tried and proved them in every rational, scientific, and available way.

We live, as Carlyle said, in a new time, a time that is full of new men; and new men are generally noisy men. In politics it is ever your very young man who is the most revolutionary. It is altogether the same in medicine, only much more so. Even the first year's medical student will speak with contempt of the white-haired general practitioner; a "G. P." he magnificently calls him. Poor goose! He does not reflect that he also will soon be a general practitioner, if, indeed, he ever rises so high; and he has not mind enough even to understand that the least famous of white-haired doctors must inevitably be much more competent at the bedside than the most brilliant of mere hospital students.

The really competent man is conscious alike of the weakness and of the strength of his professional knowledge and capacity. Knowing its weakness, he will ever keep an open eye for the new, and a prompt hand for experimentation. But knowing also its strength he will fight all his great clinical battles with his old and well-tried weapons, until he has had time and opportunity to prove the value of the new. Even then he will not cast away the old things; he will reverence and preserve them; but he will also add the new to his growing armamentarium.

